# Pleuropneumoblastome: une tumeur pédiatrique rare (à propos d’un cas)

**DOI:** 10.11604/pamj.2022.43.8.33823

**Published:** 2022-09-06

**Authors:** Rabia Yasmine Namaoui, Wissam Hadjaoui, Asma Zerabib, Leila Belbekri, Fadéla Zar, Hemana Berrah, Ali Mouats, Abdessamed Bessaïh

**Affiliations:** 1 Service d'Anatomie et Cytologie Pathologiques, CLCC Béchar, Route Ouakda, N6, Béchar, Algérie; 2 Université de Béchar Tahri Mohammed, BP 417 Route Kenadsa, Bechar, Algérie

**Keywords:** Néoplasme pulmonaire pédiatrique, syndrome DICER1, blastome pleuropulmonaire, cas clinique, Pediatric pulmonary neoplasm, DICER1 syndrome, pleuropulmonary blastoma, case report

## Abstract

Le pleuropneumoblastome est une tumeur intrathoracique rare de l´enfant, au pronostic défavorable et de diagnostic histologique. Nous rapportons ici, une observation didactique d'un enfant âgé de 3 ans qui a présenté une détresse respiratoire aigüe sévère associée à un hémothorax; l'exploration radiologique et thoracoscopique suspectaient un processus pleuro-pulmonaire malin. Ce n'est que l'examen anatomopathologique avec confrontation radio-clinique qui a permis de porter le diagnostic de pleuropneumoblastome solido-kystique de type II. Malheureusement vu la gravité du tableau clinique, l'enfant est décédé en quelques semaines à la suite d'une défaillance multiviscérale. L'expérience du pathologiste est primordiale pour le reconnaitre et initier le plutôt possible un traitement adéquat permettant une fente tumorale jusqu'à 90% en néoadjuvant et une survie à 5 ans jusqu'à au moins 53 % pour les formes agressives: solides et solido-kystiques.

## Abstract (en)

Pleuropulmonary blastoma is a rare intrathoracic tumor in children. It is associated with poor prognosis and diagnosis is based on histological examination. We conducted a didactic study involving a 3-year-old child with severe acute respiratory distress associated with hemothorax; radiological and thoracoscopic examination suggested malignant pleuropulmonary process. Anatomopathological examination with radio-clinical comparison allowed for the diagnosis of solid-cystic pleuropulmonary blastoma type II. Unfortunately, given the severity of the clinical features, the child died within a few weeks due to multiple organ failure. Pathologist experience is very important to recognize the disease and to start adequate treatment as soon as possible. This allows for a tumor regression rate up to 90% after neoadjuvant treatment and a 5-year survival rate of at least 53% for aggressive forms: solid and solido-cystic tumors.

## Introduction

Le pleuropneumoblastome (PPB) est une tumeur dysembryogenique intra thoracique rare et très agressive de l'enfant, située généralement à la périphérie du poumon mais peut être de siège pleural ou médiastinal [1-3]. Les tableaux clinique et radiologique sont peu spécifiques et dépendent du type histologique. Seule l'étude anatomopathologique permet d'évoquer le diagnostic, en montrant une prolifération biphasique: épithéliale non carcinomateuse et blastémateuse, associées ou pas à un contingent sarcomateux. Elle permet également de le classer en 3 formes: kystique, mixte (solido-kystique) ou solide dont dépend le pronostic et la prise en charge thérapeutique [1-5]. Nous rapportons ici une nouvelle observation d'un PPB de type mixte (II) chez un enfant de 3 ans.

## Patient et observation

**Présentation du patient:** le cas concerne un enfant de 03 ans, sans antécédent personnel ou familial particulier, hospitalisé en réanimation pour une détresse respiratoire sévère ayant fait suite à l´aggravation d´une toux et d´un syndrome infectieux, étiquetés initialement comme une bronchite banale trainante, non résolutive sous traitement antibiotique. Devant ce tableau clinique sévère des explorations radiologique et biologique ont été débutées.

**Figure 1:**
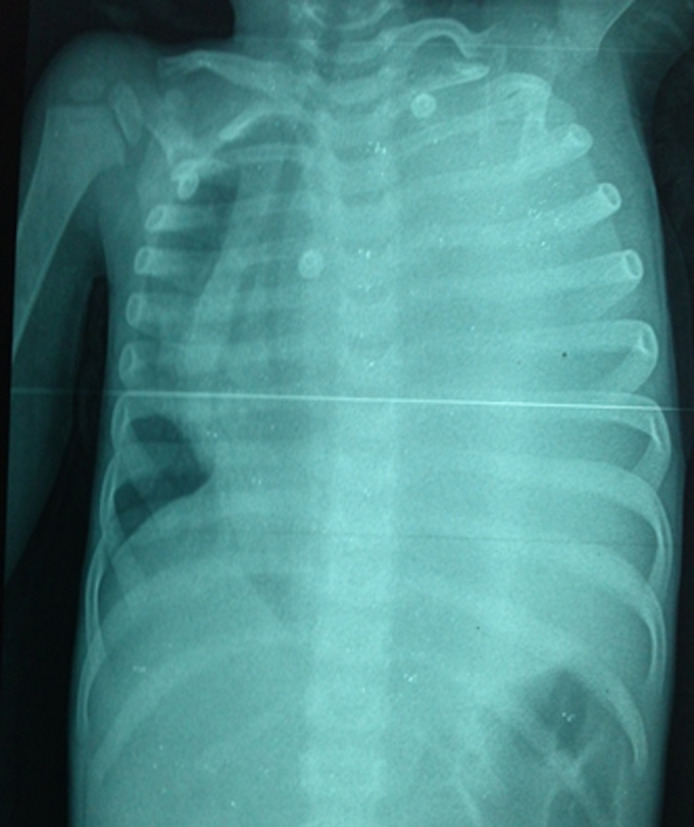
téléthorax; opacité de l’hémithorax gauche refoulant le médiastin

**Démarche diagnostique et intervention thérapeutique:** la radio thoracique a mis en évidence une opacité diffuse de l´hémithorax gauche, refoulant le médiastin (Figure 1), correspondant à un épanchement pleural liquidien de grande abondance, sero-hématique, récidivant après ponctions multiples. Le bilan biologique a montré un important syndrome inflammatoire avec une anémie modérée à 9 g/dl d'hémoglobine et des hémocultures négatives.

**Figure 2:**
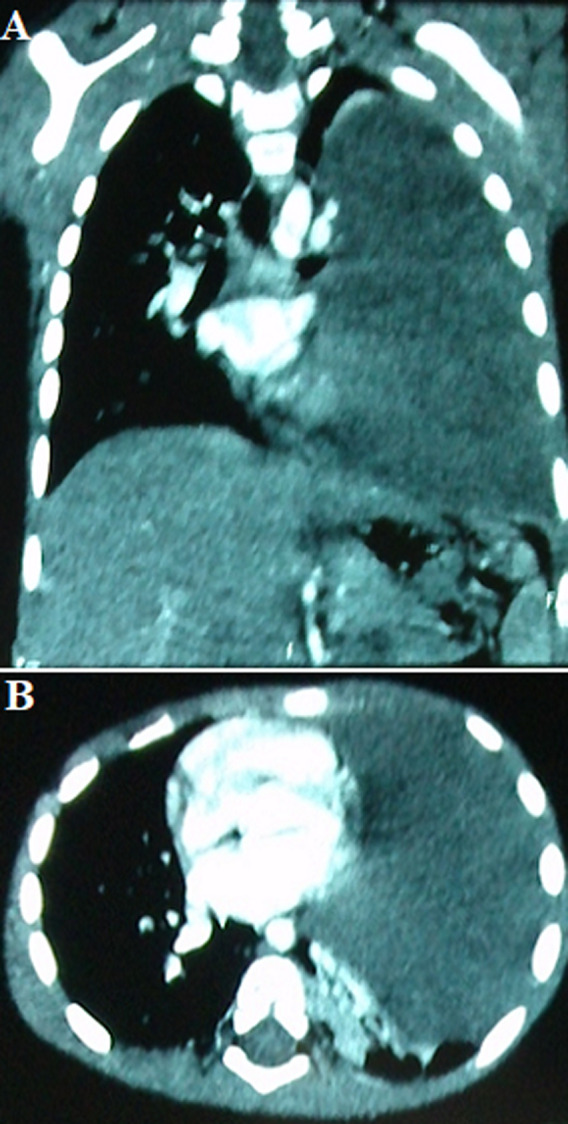
TDM thoracique montrant la présence d’une condensation pulmonaire gauche et d’un épanchement pleural liquidien cloisonné refoulant le médiastin

**Résultat et suivi:** la récidive de l'épanchement, la gravité de la détresse respiratoire et la détérioration de l'état général ont motivé la réalisation d´une TDM, d´une échographie thoracique et par la suite d'une thoracoscopie. Ces dernières ont montré respectivement après drainage; la présence d´un épanchement pleural liquidien cloisonné refoulant le médiastin ainsi qu'une condensation parenchymateuse pulmonaire, para-hilaire gauche (Figure 2 A, B), de 13 cm de grand axe, correspondant à un processus tumoral malin, solido-kystique, bourgeonnant à la thoracoscopie. Le dosage des marqueurs tumoraux, notamment les LDH, l'α Fœtoprotéine, les β-HCG et l'ACE était négatif.

**Figure 3:**
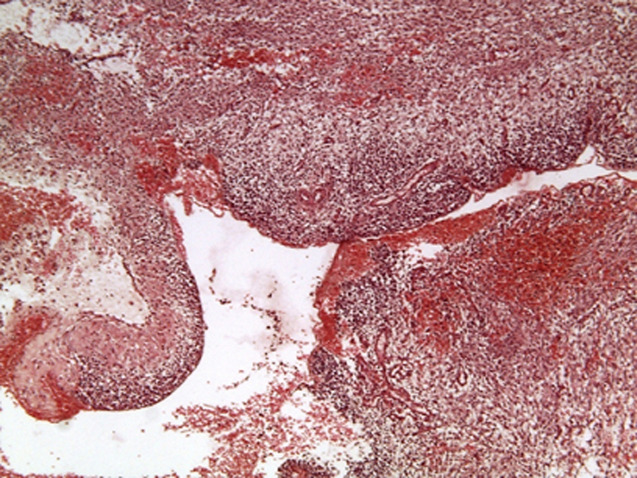
aspect microscopique au faible grandissement d'un processus tumoral mixte solido-kystique

**Figure 4:**
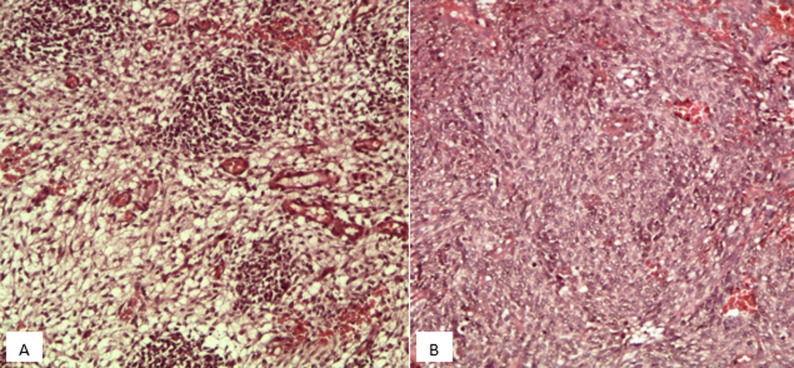
A) aspect microscopique au moyen grandissement de contingents blastémateux; B) sarcomatoïdes indifférenciés

Les biopsies réalisées lors de la thoracoscopie ont mis en évidence une prolifération tumorale maligne triphasique, solido-kystique (Figure 3), de densité cellulaire variable, avec des contingents blastémateux majoritaires, composés de petites cellules, à noyau basophile ovale, regroupées en petits amas ou dispersées au sein d´un stroma lâche myxoïde; d'un contingent sarcomatoïde fusocellulaire indifférenciés, organisés en bandes et en massifs (Figure 4) mais aussi d´un contingent épithélial minime, non carcinomateux, composé de cellules cubo-cylindriques régulières, parfois ciliées, bordant partiellement des pseudo-cavités et formant par endroits des structures tubulaires. Ce revêtement surmonte focalement des nids blastémateux, non rhabdoïdes, simulant un aspect de couche cambiale.

**Figure 5:**
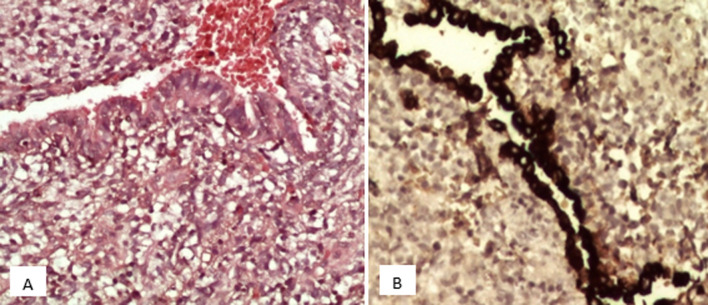
A) aspect microscopique au moyen grandissement du contingent épithélial non carcinomateux, tapissant les cavités kystiques; B) marqué par l'anticorps anti-panCK

**Figure 6:**
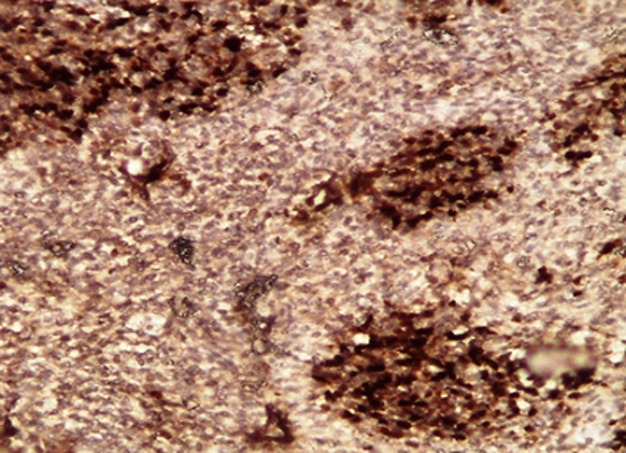
aspect microscopique de l'expression de la myogénine au niveau des nids des cellules blastémateuses

La panCK et l´EMA soulignent uniquement la composante kystique épithéliale non carcinomateuse; la composante blastémateuse exprime focalement la myogénine (Figure 5 et Figure 6) et les territoires sarcomatoïdes restent indifférenciés (Calrétinine-, CD99-, CD34-, CD31-, PS100-, AML-, Desmine-, MDM2-, NSE-, WT1-, βHCG-, PLAP- et CD45-). Ainsi le diagnostic d'un pleuropneumoblastome de type II a été posé après confrontation radio-clinique et élimination de tout processus tumoral synchrone ou métastatique extra-thoracique. Aucune étude cytogénétique n'a été par ailleurs réalisée chez le patient ou sa famille à la recherche d'une éventuelle mutation du gène DICER1. Suite au caractère agressif de la maladie et à la défaillance multiviscérale, le patient est décédé et n'a pu bénéficier d'un traitement oncologique adéquat.

**Consentement éclairé:** après des explications claires et détaillées, les parents du patient ont donné leur consentement éclairé pour la réalisation de ce travail vu que l'enfant est mineur.

## Discussion

Le pleuropneumoblastome (PPB) est une tumeur maligne rare et agressive de l´enfant, représentant 1% des tumeurs pulmonaires. Il peut être extra pulmonaire de siège pleural ou médiastinal. Il survient aussi bien chez les garçons que chez les filles avant l'âge de 4 ans. Il peut se voir en prénatal, chez l'adolescent et exceptionnellement chez l'adulte. Il est à distinguer des blastomes pulmonaires qui sont des carcinosarcomes de l'adulte. Il est considéré comme un néoplasme dysembryogénique pédiatrique, associant un contingents blastémateux, sarcomateux et une composante épithéliale non tumorale [1-5]. Il est classé en trois groupes histo-pronostiques et évolutifs: type I (kystique), type II (mixte: solido-kystique) et type III (solide). Un nouveau sous-groupe du type I est individualisé: le type I régressif/non progressif (type Ir) qui ne diffère du type I que par l'absence du contingent blastémateux; l'âge tardif de survenue et la non progression vers les formes agressives (type II et III) qui sont les plus fréquentes, représentant 80 à 85% des cas [1-3].

Son étiopathogénie reste mal connue, néanmoins 25% des PPB sont des formes familiales et seraient associés à la mutation du gène DICER1 codant pour une enzyme de la famille des Ribonucleases III, responsables de la maturation des cellules dans le thorax, le rein, l'ovaire, la thyroïde et le système nerveux, favorisant ainsi la survenue de néoplasmes dits Tumeurs du Syndrome de prédisposition familiale DICER1. D'autres anomalies sont rapportées comme la trisomie 8 et 2 ou bien, la délétion de la région 17p portant le gène TP53 [3,4]. Les manifestations cliniques du PPB ne sont pas spécifiques, font souvent retarder le diagnostic et sont corrélées à l'âge du patient et du type histologique. Le PPB peut être révélé par un pneumothorax, une toux, un syndrome infectieux ou une détresse respiratoire comme chez notre patient. Quant à l'imagerie, le type I peut être confondu avec une malformation congénitale adénomatoïde kystique ou un emphysème lobaire; seule l'histologie permet le diagnostic en montrant la présence de contingents blastémateux ou sarcomateux parfois épars au sein des parois. Néanmoins, la présence d'une mutation DICER1, de septa ou la multifocalité des lésions le suggère et impose son exérèse [1-5].

Pour les formes agressives pouvant mimer une pleuro-pneumopathie ou bien un empyème, le diagnostic est fait sur biopsies ou pièce opératoire, à condition que le pathologiste soit alerte et le différencie des métastases des autres sarcomes ou tumeurs de blastème [5], des tératomes médiastinaux mais aussi du synovialosarcome. La présence d'un contingent sarcomateux uni ou multidirectionnel fait partie de la lésion; son abondance le rend plus agressif avec tendance à l'envahissement vasculaire et aux métastases au cerveau, à l'os ou au foie. Le bilan d'extension et la surveillance permettront le dépistage des autres tumeurs synchrones ou métachrones du Syndrome DICER1. La recherche de cette mutation est systématique chez le patient et chez ses proches de premier degré [4,5].

Le traitement du PPB est avant tout chirurgical, essentiellement pour les formes kystiques dont la survie à 5 ans est estimée à 90%. La chimiothérapie complémentaire à base d'Ifosfamide, Vincristine, Dactinomycin et Doxorubicin est systématique pour les types II et III; permettant une fente tumorale jusqu'à 90% en néoadjuvant; une survie à 5 ans de 71% et 53% respectivement. La radiothérapie est réservée pour les reliquats non résécables [1-5].

## Conclusion

Bien que rare, le PPB est la tumeur maligne thoracique la plus commune en pédiatrie, particulièrement chez le nourrisson ou le petit enfant. L'absence de spécificité radio-clinique est en rapport avec les différents types histologiques. L'expérience du pathologiste est primordiale pour le reconnaitre, le différencier des autres tumeurs et le classer afin d'initier un traitement adéquat.

## Remerciements

Naïla Chamouni, Hamid Laras, Yacine Inouri, Mouloud Benaïred et Ahmed Kacimi pour leur aide et avis précieux lors de l'élaboration de cet article.
